# Grafts vs. flaps: a comparative study of Bracka repair and staged transverse preputial island flap urethroplasty for proximal hypospadias with severe ventral curvature

**DOI:** 10.3389/fped.2023.1214464

**Published:** 2023-06-21

**Authors:** Youtian Zhang, Xin Wang, Yong Wu, Shengbin Li, Dongzheng Zhang, Xiong Ma, Cong Wang, Zhenhua Zhang, Yukun Ma, Baolong Wei, Yong Guan

**Affiliations:** Department of Urology, Tianjin Children’s Hospital/Tianjin University Children’s Hospital, Tianjin, China

**Keywords:** hypospadias, Bracka repair, staged transverse preputial island flap urethroplasty, pediatric penile perception score, ventral curvature

## Abstract

**Introduction:**

Bracka repair and staged transverse preputial island flap urethroplasty are both significant methods in treating proximal hypospadias. They utilize the flap and graft techniques, respectively, to achieve a satisfactory success rate. This study aimed to compare the outcomes of these 2 methods in the treatment of proximal hypospadias with severe ventral curvature.

**Methods:**

We retrospectively analyzed 117 cases of proximal hypospadias with severe ventral curvature who had undergone either Bracka repair (*n* = 62) or staged transverse preputial island flap urethroplasty (*n* = 55). All operations were performed by a single surgeon, and the choice of method was determined by the surgeon's preference based on his experience. Cosmetic outcome was evaluated with Pediatric Penile Perception Score (PPPS). Patients' characteristics including age, penis length, glans diameter, length of the urethral defect and ventral curvature degree, cosmetic outcomes, and complication rates were all compared.

**Results:**

There was no significant difference in age, penis length, glans diameter, length of the urethral defect, or ventral curvature degree. In the Bracka group, there were 5 patients with fistula, 1 patient with stricture, and 1 case of dehiscence. In the staged transverse preputial island flap urethroplasty group, there were 4 patients with fistula, 1 with stricture, and 2 with diverticulum. The scores of shaft skin and general appearance were consistently higher in the Bracka group than in the staged transverse preputial island flap urethroplasty group. The differences in complication rate and cosmetic outcome were not statistically different (*P* > 0.05).

**Conclusions:**

Bracka repair and staged transverse preputial island flap urethroplasty are both satisfactory staged surgical options for proximal hypospadias with severe ventral curvature and have similar complication rates. Bracka repair may create a better appearance, but more studies are needed to confirm this finding. Pediatric surgeons should consider additional factors, such as the patient's specific condition, parents' inclination, and personal experience, rather than safety, to make the best choice between the 2 methods.

## Introduction

**Figure 1:**
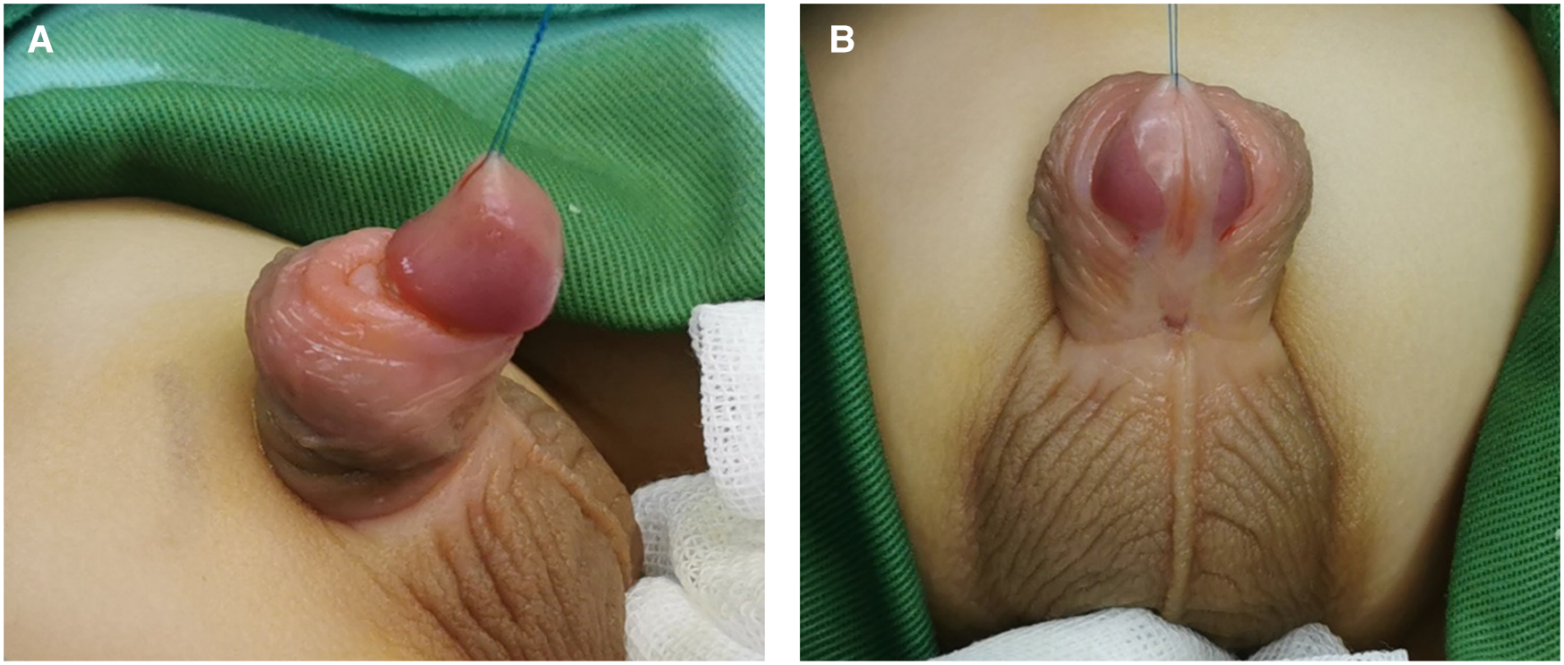
The appearance of proximal hypospadias, the ectopic meatus located at the penile scrotal junction accompanied by severe ventral curvature. (**A**) Lateral view (**B**) Ventral view.

Hypospadias is one of the most common congenital malformations of the male genitourinary system, with a reported global incidence of 0.6–34.2 per 10,000 live births (1). Hypospadias is characterized by the ectopic urethral opening being displaced along the ventral side of the penis, the only cure for which is surgery. Correction of proximal hypospadias remains a surgical challenge, which is mainly attributable to the characteristics of proximal hypospadias including a more proximal meatus, severe ventral curvature (Figure 1), and the need to transect the urethral plate during the operation (2). Although many techniques have been described to resolve this common issue, including 1-stage repair and the 2-stage repair, there is currently no definitive evidence to confirm the superiority of any technique over the others (3). Nonetheless, some studies have confirmed that 2-stage repair is a better option for proximal hypospadias with severe ventral curvature (4, 5), and a recent survey study indicated that younger surgeons prefer 2-stage techniques in treating patients with proximal hypospadias (6). Therefore, pursuing the optimal 2-stage repair that can achieve the most satisfactory functional and cosmetic outcomes for proximal hypospadias may be a valuable endeavor. Bracka repair, first described in 1995 by Bracka, is a prominent 2-stage repair that uses grafts (7). This technique has been improved over time, and recent studies have confirmed its association with an ideal success rate, especially in proximal hypospadias with severe ventral curvature (8, 9). Staged transverse preputial island flap urethroplasty (STPIF), first reported by Chen et al., is a newly adapted method using flaps based on the traditional transverse preputial island flap (TPIF) (10). STPIF has been shown to reduce the difficulty of surgery and the complication rate in proximal hypospadias treatment (11). Thus, both Bracka repair and STPIF are noteworthy 2-stage methods, and both have achieved encouraging results in the cure of hypospadias using grafts and flaps, respectively. However, to the best of our knowledge, no study has compared these 2 methods in treating proximal hypospadias. STPIF has been the option for 2-stage urethroplasty for treating proximal hypospadias in Tianjin Children's Hospital since 2017, providing us with adequate clinical data to conduct a retrospective study to compare the outcomes of Bracka repair and those of STPIF for proximal hypospadias with severe ventral curvature.

## Patients and methods

### Patients

The clinical data of patients who received initial treatment from January 2017 to August 2021 for proximal hypospadias (the location of the urethral opening was at the penile–scrotal junction or closer to the scrotum after artificial erection test) accompanied by severe ventral curvature (ventral curvature greater than 30° after artificial erection test) were retrospectively reviewed. Patients who underwent Bracka and STPIF were included in the study. We excluded patients who were admitted to our hospital for the repair of a previously failed operation. Finally, 117 patients were incorporated into our study; among them, 62 patients had undergone Bracka repair (Group A), and 55 patients had undergone STPIF (Group B). All operations were performed by a single surgeon (Dr. Guan), and the choice of method was determined by the surgeon's preference based on his experience. Information of patients regarding age, penis length, and glans diameter were measured and recorded preoperatively. Testosterone was used in patients whose glans diameter was shorter than 1.2 cm before operation.

### Surgical technique

#### Ventral curvature correction

**Figure 2:**
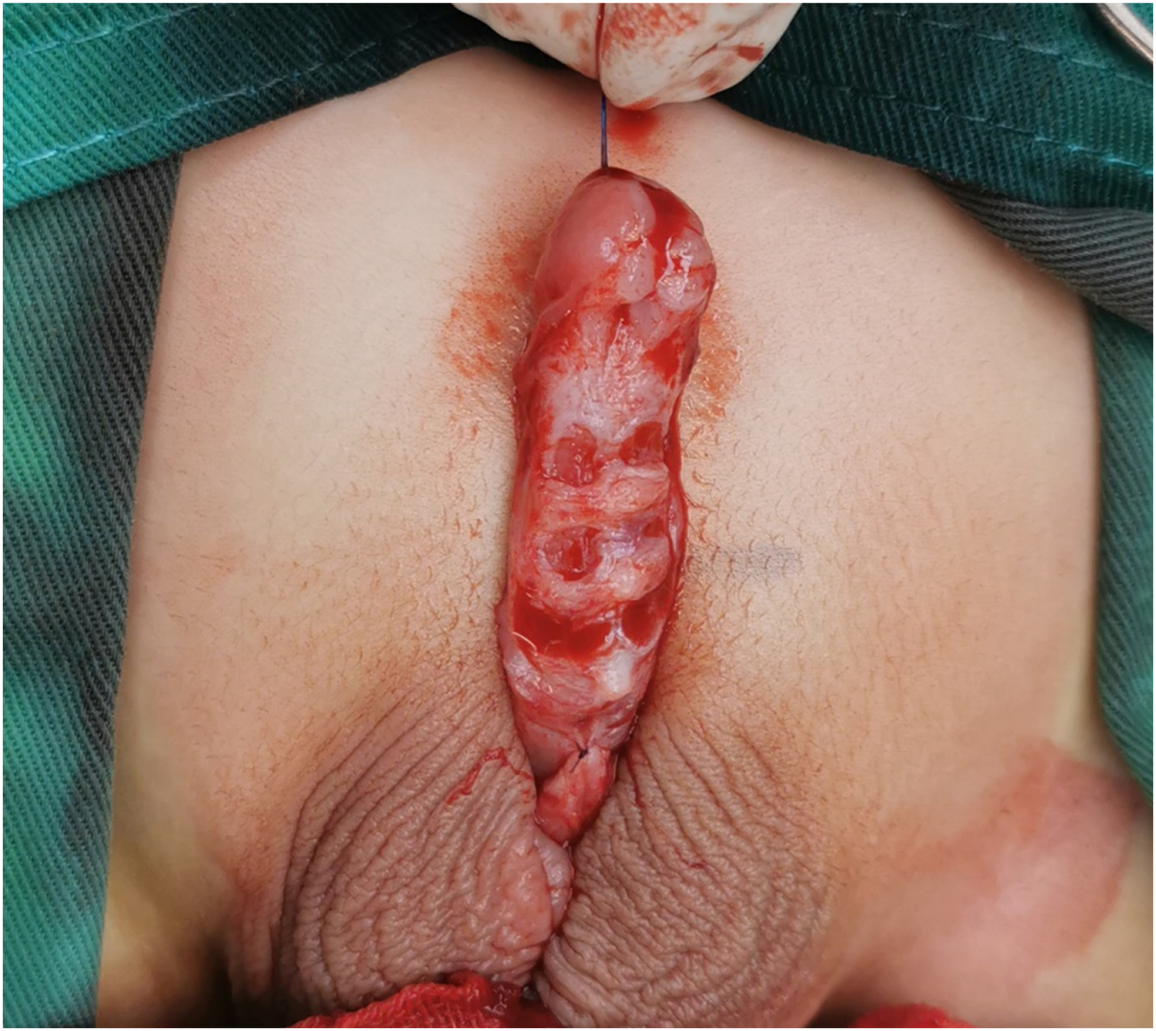
Ventral curvature correction, the penile skin was degloved, all tethering ventral bands were removed, and 3 parallel lines with a 4-mm interval on the curved area were slit.

The penile skin was degloved to the root of the penis and all tethering ventral bands were removed. Then, the degree of ventral curvature was evaluated with the artificial erection test. The urethral plate was transected if the ventral curvature was greater than 30°. If the ventral curvature remained greater than 30° after the repeated artificial erection test, the tunica albuginea was slit through a transverse line on the curved area, and 2 parallel lines marked above and below the first line, respectively, were also slit to the tunica albuginea (Figure 2).

#### Bracka repair

##### Stage I

**Figure 3:**
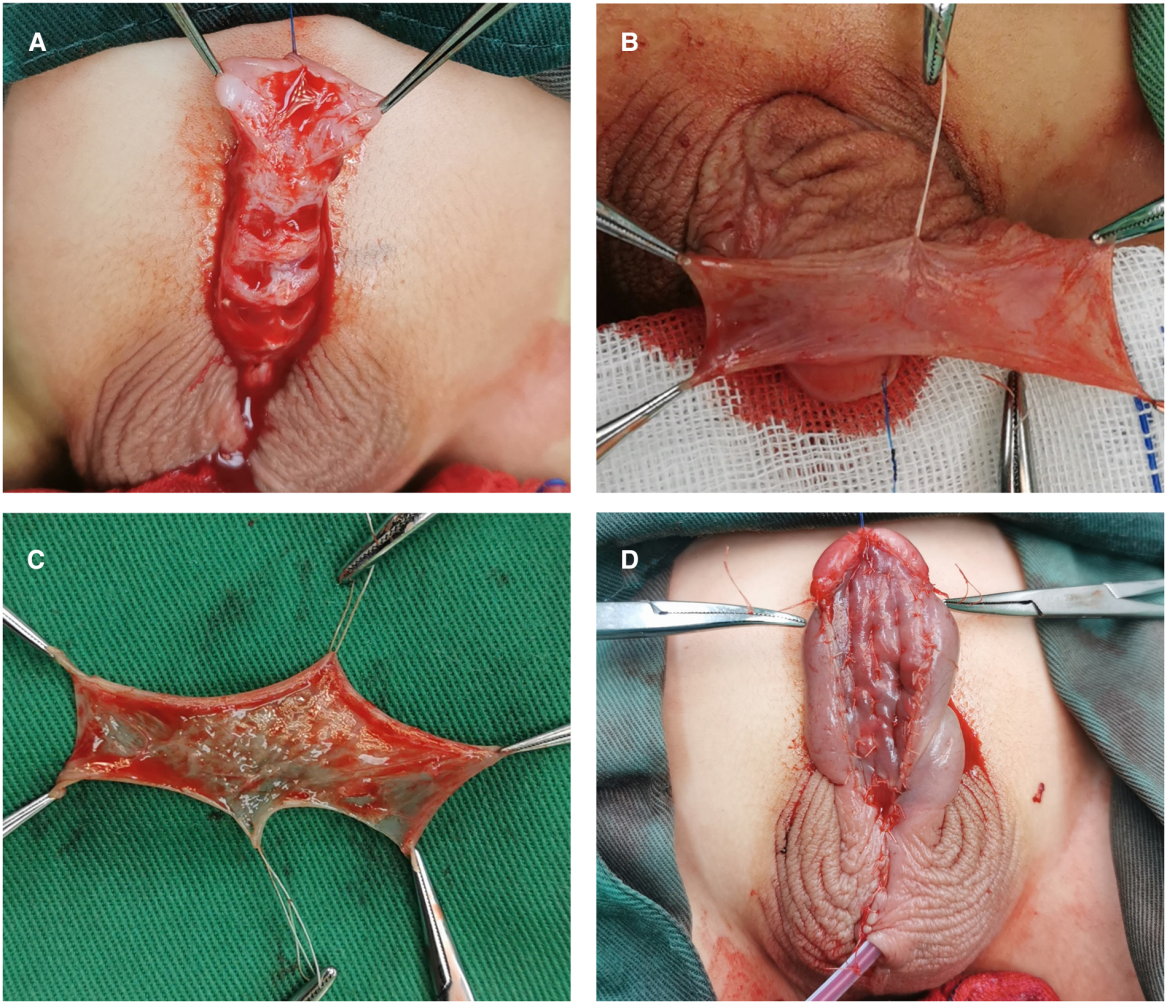
Stage I of Bracka repair. (**A**) The glans was severed, and the glans wings were unfolded to 180°. (**B and C**) A graft was designed and harvested from the inner layer of the preputial hood. (**D**) The free graft was fixed on the whole defective urethral plate by interrupted sutures.

After the ventral curvature correction, if the urethral plate was retained for a sufficient amount of lengths, proximal part of the neourethra was constructed *via* the Duplay technique. The glans was severed by incising through the midline, which was drawn from the ectopic meatus to the proposed area of the neomeatus. The wings of the glans were unfolded to 180°. A graft was designed on the inner layer of the preputial hood, the size of which was determined by the length of the urethra defect. By removing all the subcutaneous tissue, a free graft was created and was then placed in normal saline as a standby. Fixation of the free graft was performed along the whole defective urethral plate by sewing the graft to the corpus cavernosum of the ventral penis with interrupted sutures approximately 3–4 mm apart (Figure 3). After a piece of gauze on the ventral side of the penis was placed and fixed properly, a compression dressing was used for the transplanted graft strip. At the end of the surgery, a urinary catheter was placed through the ectopic meatus.

##### Stage II

**Figure 4:**
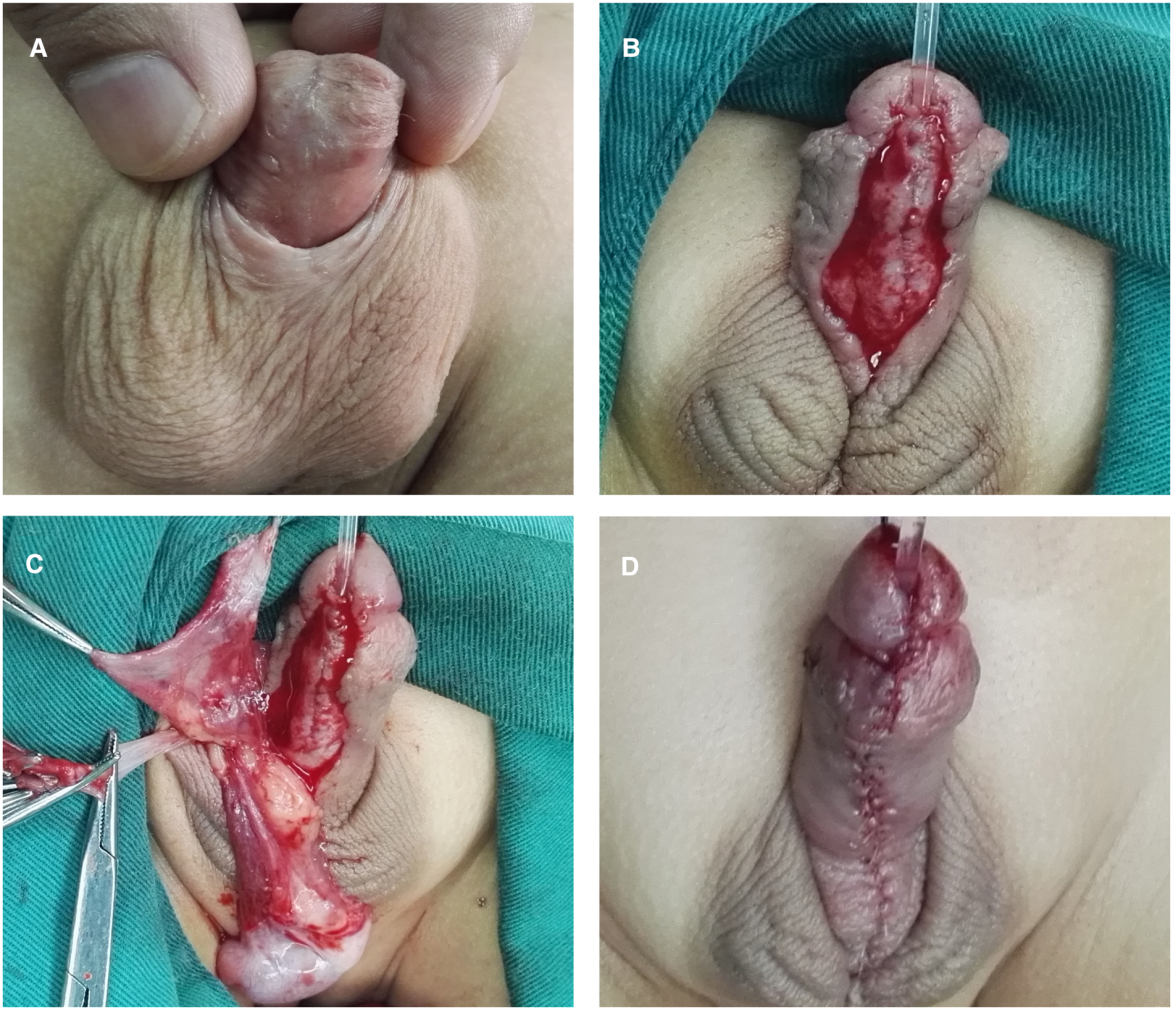
Stage II of Bracka repair. (**A**) The appearance of the penis 6 months after the first stage. (**B**) The new urethral plate strip was tubed over a catheter using continuous extraluminal inverting sutures. (**C**) The tunica vaginalis from a testicle was dissociated to create a waterproofing layer. (**D**) The appearance of the penis after the second stage.

The second stage was performed 6 months later when the graft had become a smooth and well vascularized new urethral plate. An artificial erection test was repeated to exclude stubborn ventral curvature. The U-shaped incision lines, which delineated a 1.5-cm wide strip, were drawn for the subsequent tubularization. The new urethral plate strip was tubed over a catheter by continuous extraluminal inverting sutures. The tunica vaginalis from a testicle or dartos from the scrotum were dissociated to create a waterproofing layer. The glans was repaired by reconstructing the glans spongiosum with sutures, which was followed by glans epithelium closure with simple interrupted sutures (Figure 4). After the penile skin was sutured, the penis was dressed, and a urethral catheter was retained for urinary diversion.

#### Staged transverse preputial island flap urethroplasty

##### Stage I

**Figure 5:**
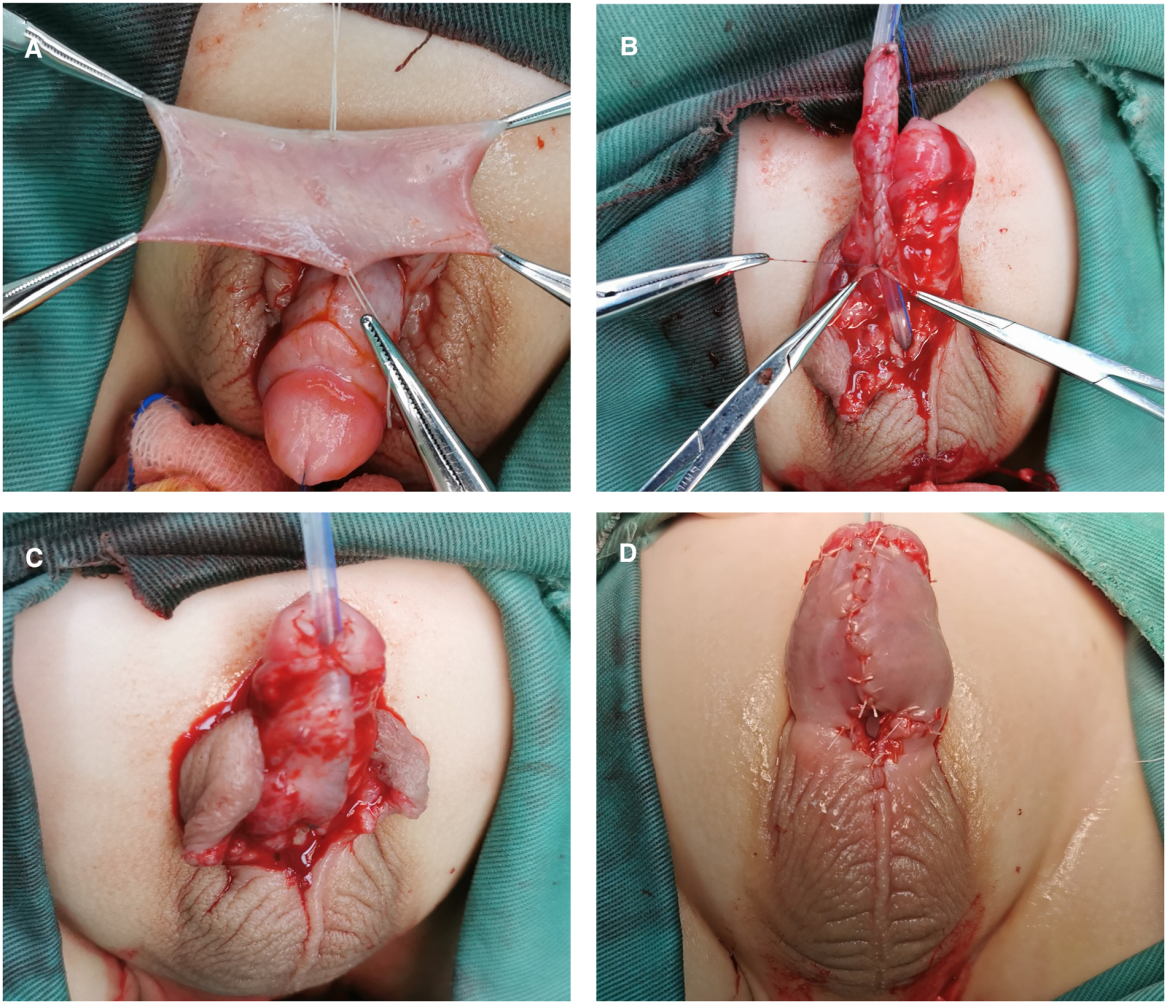
Stage I of staged transverse preputial island flap urethroplasty. (**A**) A transverse rectangular flap was dissected from the inner plate of the dorsal prepuce. (**B**) The flap was tubularized over a silicone catheter with continuous sutures and transposed ventrally. (**C**) The distal end of the neourethra was sutured with the glans tip. Byar's flaps were created and transposed to cover the neourethra and ventral defect. (**D**) The appearance of the penis after the first stage, a fistula remained on the ventral side of the penis.

According to the distance between the ectopic meatus and the glans tip, a transverse rectangular flap was dissected from the inner layer of the dorsal prepuce. The width of the flap was around 12 mm. The vascular pedicle was proximally divested to the root of the penis. The flap was then tubularized over a silicone catheter with continuous sutures. The glans was severed by incising through the midline, and both sides were unfolded extensively. The neourethra made by the flap was transposed ventrally, running parallel to the penile long-axis direction. The distal end of the neourethra was sutured with the glans tip to form a new urethral orifice, and then the 2 wings of the glans were closed. After this, the proximal end of the neourethra was sewn to the underlying corporal body. Byar's flaps were created using a dorsal prepuce and transposed to cover the neourethra and ventral defect. Consequently, a neourethra replaced the pre-operative urethral plate, the near end of the neourethra and the ectopic meatus were left on the ventral side of the penis, shaped like a fistula. In the end, a silicon indwelling catheter was retained. (Figure 5).

##### Stage II

The second stage procedure was performed 6 months after the first procedure. An annular incision reaching the Buck's fascia was designed to extend from the artificial fistula up to the distal neourethra. The closure of the artificial fistula was completed by double-layer inverting sutures with a silicon catheter as support. In the end, the penis was dressed, and a urethral catheter was retained for urinary diversion in the same manner as that described for Bracka repair.

### Postoperative care

Intravenous antimicrobial treatment was administered for 1 day, the dressing was retained until 1 week after the operation, and the urinary catheter was removed after 12 postoperative days in both groups. All parents were advised to give their children a hip bath to promote wound healing.

### Follow-up

A routine follow-up scheme was executed in all cases, which consisted of regular visits 1 week, 2 months, 6 months, and 1 year after the operation, and then yearly follow-up visits were suggested to parents. Complications were recorded during this period, including recurrence of ventral curvature, urinary fistula, stricture, glans dehiscence, and diverticula formation. When patients visited the clinic 6 months after the operation, the cosmetic results were assessed by the parents using Pediatric Penile Perception Score (PPPS) (12). We provided a paper PPPS (Chinese version) to parents and invited them to complete it on the spot. The surgeon and a surgical peer who did not participate in the operation were also asked to evaluate the appearance using the PPPS. The scores of each patient derived from 3 parties were recorded in the medical record after evaluation.

### Statistical analysis

All the aforementioned information was collected from the hospital medical records. Mean values (±SD) are used to describe patients' preoperative characteristics (age, penis length, glans diameter, length of urethral defect, ventral curvature degree), duration of follow-up, and the PPPS. The Student *t* test was used to compare the data between groups. The usage of transverse corporotomies and complication rate are expressed as the number of cases with percentages, and the *χ*^2^ test was used for comparisons between groups. All data analysis was performed using SPSS 21.0 (IBM Corp., Armonk, NY, USA), and *P* < 0.05 was considered statistically significant.

## Results

### Characteristics of patients

A total of 117 patients were integrated into our study. Among them, 62 patients had undergone Bracka repair (Group A) and 55 patients had undergone STPIF repair (Group B). The surgery age in Group A ranged from 10 months to 38 months (mean age 26.73 months), while the surgery age in Group B ranged from 9 months to 36 months (mean age 25.27 months). The follow-up period ranged from 12 to 40 months (mean follow-up 19.63 months) in Group A and from 11 to 35 months (mean follow-up 20.42 months) in Group B. Other characteristics of patients are summarized in Table 1. No statistically significant differences were found in these data (*P* > 0.05).

**Table 1:** Characteristics and follow-up duration of patients.

Variables	Group A	Group B	*P*-Values
Age (month)	26.73 ± 5.28	25.27 ± 5.56	0.15
Penis Length (cm)	1.26 ± 0.21	1.21 ± 0.24	0.23
Glans Diameter (cm)	3.46 ± 0.23	3.39 ± 0.25	0.12
Urethral Defect (cm)	3.44 ± 0.21	3.37 ± 0.24	0.10
Curvature Degree (°)	56.68 ± 10.21	59.16 ± 9.97	0.19
Follow-up (month)	19.63 ± 5.57	20.42 ± 4.18	0.39
Transverse corporotomies *n* (%)	37 (59.7%)	34 (61.8%)	0.81

### Complication rates

No complications were observed after the first stage in both groups. Postoperative complication rates after the second stage were 11.3% and 12.8% in Group A and Group B, respectively. All patients received urethral plate dissection since the ventral curvature was greater than 30° in every case. No recurrence of ventral curvature occurred in either group. Fistula was observed in 5 patients (8.1%) in Group A and 4 patients (7.3%) in Group B. All of them were treated successfully with fistula repair. After the second-stage operation, 1 patient in each group (1.6% in Group A, 1.8% in Group B) had urethral stricture. After receiving urethral dilation, both patients achieved satisfactory improvement. There was 1 case (1.6%) of glans dehiscence in Group A and none in Group B. Dehiscence repair was performed successfully in this case. There were 2 patients (3.6%) in Group B who developed diverticulum and received diverticulectomy; however, no cases of diverticulum were observed in Group A. The statistical comparison of complication rate and success rate is shown in Table 2. Although the incidence of each complication was different in Group A and Group B, none of the differences were statistically significant.

**Table 2:** Comparison of complication rate and success rate.

Variables	Incidence *n* (%)		*P*-Values
	Group A (*n* = 62)	Group B (*n* = 55)	
Fistula	5 (8.1%)	4 (7.3%)	>0.99
Stricture	1 (1.6%)	1 (1.8%)	>0.99
Dehiscence	1 (1.6%)	0	>0.99
Diverticulum	0	2 (3.6%)	0.22
Success rate, %	88.7	87.2	0.81

### PSSS score

The PPPS scores of patients in both groups evaluated by 3 parties (parents, surgeon, surgical peer) were calculated and are presented in Table 3. In the meatus item, the surgeon and surgical peer gave higher marks to Group A. Scores of Group B from parents were slightly higher. In the glans item, the surgeon gave the higher marks to Group A; however, parents and the surgical peer indicated that the glans in Group B had a better appearance. Interestingly, for the shaft skin and general appearance, scores of Group A from all 3 parties were higher than those from Group B. However, the differences were not statistically significant.

**Table 3:** Comparison of average PPPS scores judged by parents, the surgeon, and one surgical peer.

Item	Judge	Group A	Group B	*P*-Values
Meatus	Parents	2.27 ± 0.32	2.35 ± 0.25	0.14
	Surgeon	2.56 ± 0.34	2.47 ± 0.31	0.14
	Surgical peer	2.48 ± 0.29	2.40 ± 0.33	0.17
Glans	Parents	2.03 ± 0.43	2.11 ± 0.36	0.28
	Surgeon	2.56 ± 0.39	2.49 ± 0.35	0.31
	Surgical peer	2.19 ± 0.41	2.24 ± 0.44	0.53
Shaft Skin	Parents	2.10 ± 0.45	1.96 ± 0.42	0.09
	Surgeon	2.49 ± 0.33	2.38 ± 0.34	0.08
	Surgical peer	2.28 ± 0.31	2.18 ± 0.32	0.09
General Appearance	Parents	2.21 ± 0.34	2.09 ± 0.36	0.07
	Surgeon	2.53 ± 0.38	2.40 ± 0.35	0.06
	Surgical peer	2.39 ± 0.42	2.25 ± 0.39	0.07

## Discussion

With the progress of technology and the accumulation of clinical experience, pediatric urologists have made great advances in the management of hypospadias; nevertheless, it is worth noting that most of these have been made in the management of distal hypospadias (3). A recently published review frankly points out that the incidence of postoperative complications in proximal hypospadias is higher than that assumed (13). Proximal hypospadias not only involves the posterior ectopic meatus but is also often accompanied by a smaller glans, more defective penile skin, and more severe ventral curvature; all these factors make the surgery for proximal hypospadias a challenging task. However, the results of many studies have indicated that staged repair may provide hope for tackling this problem. Christopher et al. retrospectively reviewed 167 consecutive boys undergoing repair for proximal hypospadias and found that the high complication rates in proximal hypospadias were mainly attributed to 1-stage repair (14). Other analogous studies also reported that the 2-stage repair of proximal hypospadias achieved better results compared to 1-stage repair (15, 16). Based on these findings, persisting in discussing and comparing various staged methods to pursue the optimal approach is critical for patients with proximal hypospadias. Since first being described in 1995, Bracka repair has been well received as a staged repair for proximal hypospadias (2). This method makes full use of the graft's advantages and, for example, can provide good fixation of the neourethra to the corpora, thereby solving the issue of diverticulum caused by the flap technique in 1-stage repair (7). STPIF is a relatively new method that improves upon TPIF, which was first described by Duckett in 1980 (17). TPIF had been an efficacious 1-stage method for treating proximal hypospadias with severe ventral curvature, but as time went on, problems with this once revolutionary technology emerged, for instance, a high incidence of anastomotic stenosis and the requirement of considerable clinical experience (5). To fully exploit the advantages and bypass the disadvantages of TPIF, STPIF was described by Chen et al. in 2016 (10). Leaving the ectopic urethral orifice as a fistula for the next stage of surgery to improve the effect of urethroplasty is a significant concept for hypospadias repair. Duplay recommended 3 steps or stages of repair in the 19th century. First, excision of chordee, second, neourethra tube reconstruction, third, anastomosis of the neourethra to the proximal native urethra (18). STPIF retains the original hypospadiac meatus and neourethra created by tubed flaps after the first stage, giving the neourethra a more stable environment to mature. For one, because urine is excreted through the ectopic meatus, it avoids the adverse effects of metabolic substances in urine on wound healing; for another, it diminishes the impact of the pressure of urine flow on the neourethra. Consequently, STPIF could reduce the occurrence of complications that tend to occur in TPIF, such as urinary fistula and urethral stricture (10). As the core of a variety of techniques, flaps and grafts were compared and discussed in proximal hypospadias by Powell et al. in 2000 (19). However, since the 2-stage technology was not mature at that time, the study only focused on the 1-stage repair to compare flaps and grafts. In view of the above, it is clinically significant to compare the clinical presentation of these 2 methods in the treatment of proximal hypospadias since they represent the significant usage of grafts and flaps in 2-stage repair for proximal hypospadias. To the best of our knowledge, no previous study has made such a comparison.

Severe ventral curvature often appears in proximal hypospadias, and it is usually handled as the first step of surgery. Mild-to-moderate ventral curvature could be corrected after degloving the penile skin and removing the ventral fibrous band, but severe ventral curvature needs further treatment. The current consensus suggests that urethral plate transection supplemented with dorsal plication is an effective means for treating severe ventral curvature in proximal hypospadias (20). Snodgrass introduced a method using transverse ventral corporotomies (21). He indicated that slitting 3 parallel lines with 4-mm intervals on the curved area is effective for correcting the obstinate curvature. This conclusion informs our practice, in this study, transverse corporotomies was performed in 37 patients (59.7%) in Group A and 34 patients (61.8%) in Group B, no recurrence of ventral curvature was found in our study.

Urethrocutaneous fistula, which is related to many factors such as a lack of blood supply, inadequate urinary diversion, and a low-quality waterproof layer, is the most common complication after hypospadias repair (22). In the present study, fistula was seen in 8.1% of patients in Group A and 7.2% of those in Group B. Other authors have reported an incidence of fistula from 3.4% to 21% with Bracka repair (4, 8, 23–25), and the reported incidence of fistula following STPIF ranges from 4.2% to 7.8% (5, 10, 11, 26). It appears that the incidence of fistula is more variable in Bracka repair than in STPIF. However, we believe that these figures do not imply that the STPIF is a more stable method, as fewer studies have been conducted on STPIF than on Bracka repair. The longer a technique has been in existence, the greater the variability in success rates reported in the literature. This is because as increasing numbers of surgeons use this technology, individual ability and experience become important variable that cannot be ignored. In our study, 2 different types of surgery were performed by the same surgeon, and we observed almost the same incidence of fistula in both groups.

Postoperative urethral stricture is also still among the most complex conditions to treat in hypospadias (27). In our study, 1 patient in each group experienced stricture. The overall stricture rate of Group A (1.6%) was lower than the 7% reported in Bracka's paper (7). However, considering that most of the strictures were of late onset in Bracka's study, and his patients included salvage cases, our results may only represent the short-term effect of patients after their first surgery. After all, our duration of follow-up was shorter and we excluded patients who had undergone a failed operation. The stricture rate of Group B was 1.8%, which is close to the 2.4% reported by Chen et al. (10). Lin et al. reported that no urethral stricture occurred in any of the patients who had undergone STPIF in their study, and they attributed this result to the prolonged dwelling time of the urinary catheter (3 weeks) (11). However, considering that the difference in the incidence of stricture is not significant, and taking care of pediatric patients with the urinary catheter is a challenge for parents, more research is needed to confirm the benefit of prolonging the indwelling time of urinary catheter.

Glans dehiscence often results in a deformed glanular meatus and may lead to spraying upon urination. The proximal meatal location is closely linked to this complication (28). In our study, 1 patient with glans dehiscence was observed in Group A, and no cases of glans dehiscence were observed in Group B, but the incidence was not statistically significant. We believe that the 2 methods treated the glans equally, leading to the approximately identical outcomes. The glans construction in STPIF for our study did not adopt the channel technology described by Chen et al. (10); rather, the glans was slit as in Bracka repair; according to Snodgrass' (29), severing the glans more extensively could reduce the risk of glans dehiscence.

Hypospadias repair is the main cause of the acquired diverticulum in children. This complication often occurs due to poorly supported tissue or distal stricture (30). The incidence of the diverticulum was higher in Group B in the present study. Diverticulum occurred in 3.6% of patients in Group B, and there were no cases of diverticulum in Group A. We speculate that this may be attributable to the unique characteristics of tubularization technology. Although STPIF mitigates the impact of the high pressure of urine flow on the distal neourethra and provides the neourethra sufficient time to form a more stable connection with the corpora, the final reconstructed urethra is still composed of flaps. The strength offered by the combination of the corpus cavernosum penis and tube made by flaps is inevitably weaker than that achieved by grafts. The latter is generally tightly stitched onto the corpus cavernosum penis after being fully unfolded, which enables a larger contact area and firmer connection. However, since the difference in rate did not reach statistical significance, we believe that studies with a larger sample size are needed to clarify whether there is a difference in diverticulum rate between the 2 methods.

Since modern surgical techniques have reduced the complication rate, cosmetic outcome has gradually risen in prominence as a concern in hypospadias repair (31). To date, there are no standardized algorithms for the evaluation of cosmetic outcome after hypospadias repair (32). We selected PPPS as the evaluation tool in our study since it is concise and has been shown to have a positive concordance (12). In order to be as objective as possible, we asked patients' parents, the surgeon, and a surgical peer to evaluate the cosmetic outcome of each patient. After statistical calculation, the results of meatus and glans items did not show a consistent trend. However, scores of shaft skin and general appearance were higher in Group A than in Group B, and the trend was consistent among the 3 parties. We consider these differences might be due to the technical characteristics of the 2 methods. In STPIF repair, flaps needed to be transferred to the ventral side of the penis to construct the neourethra. The tension of any twist could cause asymmetry in the appearance of the skin. As for Bracka repair, part of the neourethra was constructed in stage I. The second stage was equivalent to repairing the mild hypospadias without ventral curvature, and the new urethral plate was smooth and wide, which provided the ideal and sufficient material for urethroplasty. To sum up, it might be easier for creating the desired appearance in Bracka repair. Although the Bracka procedure might achieve a better appearance in the aggregate according to scores, the differences were not significantly different. We believe that additional research from multiple perspectives is needed to comprehensively compare the cosmetic outcomes between these 2 methods, as the evaluation of appearance is highly subjective.

As mentioned above, the surgical treatment effect was similar between the Bracka repair and STPIF. There was no statistical evidence to confirm that either technique is more appropriate than the other for proximal hypospadias with severe ventral curvature in the present study. Despite the lack of statistical difference in safety between the 2 methods, we believe each has unique advantages. Bracka repair has a wider scope of application since the source of grafts is not limited to the foreskin. A recent retrospective study compared the treatment results of Bracka repair using preputial vs. buccal grafts in 220 patients with proximal hypospadias; the authors found that the functional results were nearly the same, and the cosmetic results were even better in the buccal mucosa group (24). Mitsukawa et al. reported that they performed a modified Bracka repair using oral mucosal grafts for 6 severe proximal hypospadias cases, and satisfactory results were obtained in all the patients (33). Therefore, when a patient does not have ample foreskin, or if his penile skin has multiple scarred areas due to a previous operation, Bracka repair is undoubtedly a better choice. Moreover, Bracka repair may yield a better appearance according to our results. However, only a small portion of the neourethra was shaped in the first stage of the Bracka repair, and major defects were left after the first operation. According to our clinical experience, many parents may consequently endure a considerable psychological burden during the interval between the 2 operations. As for STPIF, the main surgical procedure is completed in the first stage, the penis could possess an acceptable appearance after the first operation, and the second stage is equivalent to a fistula repair. These factors would enable parents to maintain a more positive mindset while waiting for the second surgery. For the sake of diminishing the trauma experienced by the children, parents also may be more willing to accept STPIF.

The major limitation of this study is that it was conducted retrospectively. The grouping of patients was not completely random. Hence, the surgeon's individual preference might have led to statistical bias. As the duration of follow-up was relatively short, certain long-term complications might have been missed. A prospective study with a longer follow-up duration will provide more powerful evidence for the comparation of these 2 methods.

Our research provides a clinical reference for pediatric surgeons to select the most appropriate method for proximal hypospadias with severe ventral curvature. Our future research will concentrate on comparing the long-term results between Bracka repair and STPIF. We also believe that voiding function and psychological health, among other factors, should be included in subsequent research to more comprehensively evaluate both methods.

## Conclusion

Both Bracka repair and STPIF are reliable staged surgical options for treating proximal hypospadias with severe ventral curvature, as both could achieve a satisfactory success rate. The incidence of residual ventral curvature, urethrocutaneous fistula, urethral stricture, diverticulum, and glans dehiscence was similar between the 2 methods in this study. Although the cosmetic outcomes of Bracka appeared to be better, the difference was not statistically significant. Pediatric surgeons should consider more factors other than safety, including patient's specific conditions, parents’ inclination, and personal experience, to make the best choice between the 2 methods.

## Data Availability

The original contributions presented in the study are included in the article, further inquiries can be directed to the corresponding author.
